# Current Mood vs. Recalled Impacts of Current Moods after Exposures to Sequences of Uncertain Monetary Outcomes

**DOI:** 10.3389/fpsyg.2017.00066

**Published:** 2017-01-26

**Authors:** Lars E. Olsson, Tommy Gärling, Dick Ettema, Margareta Friman, Michael Ståhl

**Affiliations:** ^1^Samot - The Service and Market Oriented Transport Research Group, Karlstad UniversityKarlstad, Sweden; ^2^Department of Psychology, University of GothenburgGothenburg, Sweden; ^3^Department of Human Geography and Planning, Utrecht UniversityUtrecht, Netherlands

**Keywords:** sequence of events, instant utility, remembered utility, emotional response, current mood

## Abstract

Events in a sequence may each be evaluated as good or bad. We propose that such good-bad evaluations evoke emotional responses that change current mood. A model of recurrent updating of current mood is developed and compared to a model of how a sequence of events evoking emotional responses is evaluated retrospectively. In Experiment 1, 149 undergraduates are presented sequences of lottery outcomes with a fixed probability of losing or winning different amounts of money. Ratings of current mood are made after the sequence. Retrospective evaluations are either made after the ratings of current mood or, in a control condition, when no ratings of current mood are made. The results show an expected effect on current mood of the valence of the end of the sequence. The results are less clear in showing an expected beginning effect on the retrospective evaluations. An expected beginning effect on retrospective evaluations is found in Experiment 2 in which 41 undergraduates are first asked to remember the different amounts of money, then to evaluate the sequence as lottery outcomes.

## Introduction

A choice frequently results in a sequence of outcomes extended in time. Everyday examples include making a journey, reading a novel, viewing a movie, or listening to a concert. Other examples from professional life are investors encountering returns on investments, doctors prescribing multiple medical treatments or researchers conducting multiple studies. In this article, we ask how evaluations are made of sequences of events that may or may not be outcomes of choices, and when and how the evaluations evoke emotional responses.

A relevant distinction is made by Schreiber and Kahneman (2000) between instant utility and remembered utility. Instant utility is an evaluation of each event in a sequence. Remembered utility is the retrospectively aggregated evaluation of the sequence of instant utilities. In previous research (e.g., Ariely, 1998; Schreiber and Kahneman, 2000) an evaluation has been assumed to be identical to an emotional response. Yet, an evaluation that has a hedonic tone does not invariably evoke an emotional response (Russell, 2003). Therefore, we distinguish an evaluation of a choice outcome as good or bad (instant utility) from an emotional response to the outcome. A comedy movie may be funny but may not evoke an emotional response unless the evaluation has bearings on the viewer's reality. Sad music may be perceived as such without making the listener feel sad if it is no threat to well-being.

In this paper our aim is to investigate the emotions people experience during a sequence of events and their memory of these emotions. We assume that people invariably experience a non-transient background emotion referred to as current mood (Diener et al., 2015). Occasionally an event evokes an emotional response experienced against this background (Lazarus, 1991). In one of two models we propose that emotional responses to events influence current mood through a process of recurrent updating. A reported current mood after a sequence of events is therefore a temporary state in this updating process. In the second model, recall of the updated current moods is the result of reconstruction from memory of the instantaneous impacts on current mood of the emotional responses. A retrospective evaluation of the sequence is made by averaging the recalled mood impacts. In the next two sections we present these two models followed by hypotheses derived from the models. We then report two experiments to test the hypotheses.

## Current mood

Russell (2003) posits that core affects are elemental building blocks involved in all moods and emotions. More precisely, a core affect is a “neurophysiological state consciously accessible as the simplest raw (non-reflective) feelings evident in moods and emotions” (p. 148). Core affects are always accessible, either being neutral or having any other value in a dimensional system defined by the orthogonal axes pleasure-displeasure (valence) and activation-deactivation (activation) (Russell, 1980, 2003; Yik et al., 2011; Kuppens et al., 2013). Several different methods to measure affect [self-reports, pheripheral physiology, startle responses, electroencephalography (EEG) or face expressions measured with electromyography (EMG)] support a dimensional description although not all methods converge on the two dimensions of valence and activation (or arousal) (Mauss and Robinson, 2009). Additional corroboration of the valence and activation dimensions comes from neuro-imaging research (Posner et al., 2009; Wilson-Mendenhall et al., 2013).

Russell (2003) defines mood as a prolonged core affect that presumably is both influenced by changes in internal states (e.g., bodily changes, ruminations, or similar mental contents) and by emotional responses to external events (e.g., Verduyn et al., 2009, 2011). The changes in mood are likely to be less transient and weaker than the emotional responses. We propose that current mood (a self-assessment of the form “how I feel now”) is a non-transient emotion that is recurrently and instantaneously updated if the evaluations of events (instant utilities) evoke emotional responses.^1^ This is represented by the following equation relating current mood (*CM*_*i*_) at time *i* (ϵ 1,…, n) to current mood at time *i*–1 and the instant utility (*IU*_*i*_) of an event at time *i* varying on a single continuum *X*,

(1) $$\text{C}M_{i}=\{\begin{array}{l} CM_{i-1}+\left(CM_{max}-CM_{i-1}\right)\left(1-\exp\left(-\phi_{i}IU_{i}\right)\right)\\ \;IU_{i}\geq 0;0\leq \phi_{i}\leq 1\\ CM_{i-1}+\left(CM_{i-1}-CM_{min}\right)\left(1-\exp\left(\phi_{i}IU_{i}\right)\right)\\ \;IU_{i}<0 \end{array}$$

The instant utility *IU*_*i*_ has an impact on *CM*_*i*_ that depends on ϕ_*i*_ varying from no impact (0) if the instant utility does not evoke an emotional response to an impact that is equally large as the instant utility (1). The degree of change is limited by *CM*_*max*_ = −*CM*_*min*_ > 0.^2^ We further propose that the instant utility (*IU*) is represented by prospect theory's value function (Kahneman and Tversky, 1979; Tversky and Kahneman, 1992; Carter and McBride, 2013), where *c* is an adaptation level (Baucells et al., 2011) which determines whether *X* is evaluated as good or bad, *a*_*G*_ a weight placed on a good evaluation (*X*–*c* > 0), *a*_*B*_ a weight placed on a bad evaluation (*X*–*c* < 0), and *b* a curvature parameter,

(2) $$IU=\{\begin{array}{c} -a_{B}{\left|X-c\right|}^{b}\;\;c<X;\;a_{B},\;b>0\\ a_{G}{\left(X-c\right)}^{b}\;\;c\leq X;\;a_{G},\;b>0 \end{array}$$

In prospect theory, the instant utility (*IU*) is a non-linear function of *X* (0 < *b* < 1) and a bad evaluation has a stronger impact of *X* than a good evaluation has (a_*B*_ > a_*G*_).

Note that equations 1 and 2 imply that mood is unidimensional, whereas mood was above defined as a prolonged core affect that varies in both valence and activation. Kron et al. (2015) found that physiological indicators of emotions elicited by visual stimuli were related to valence but only weakly to activation, and since previous research has demonstrated a weak positive linear correlation (e.g., Västfjäll and Gärling, 2007), valence and activation may be combined additively to a single dimension that varies from positive valence and high activation to negative valence and low activation. This corresponds to a dimension in the affect grid ranging from elation to disappointment (Västfjäll and Gärling, 2002, 2006), implying that valence is amplified by activation (Kahneman and Miller, 1986; Mellers, 2000).

## Recalled mood impacts

A question people may ask retrospectively is “How did I feel in the recent past?” This is also the question researchers ask participants in studies of emotional well-being (e.g., Diener and Lucas, 2000). It has been proposed that such retrospective evaluations depend on the aggregation of instant utilities (Kahneman, 2000a,b). Several studies have demonstrated a peak-end aggregation rule (e.g., Ariely, 1998; Schreiber and Kahneman, 2000; Redelmeier et al., 2003; Langer et al., 2005; for review see Fredrickson, 2000). According to this rule the final outcome (end) and the most intense outcome (peak) are averaged. The rule is thus a special case of a general averaging rule which has been contrasted to an additive rule (e.g., Cojuharenco and Ryvkin, 2008; Seta et al., 2008a,b). Below we propose a weighted averaging rule.

For a demarcated sequence of events (e.g., a journey, a concert) that evoke emotional responses such that current mood changes, the evaluation of the sequence may depend on an aggregation of the recalled moods during the sequence. This would be consistent with our model of how mood changes with emotional responses evoked by evaluations of the events in the sequence. However, the argument has been empirically supported that emotions are only possible to reconstruct from memory of their determinants (Robinson and Clore, 2002a,b). The implication is hence that people are not able to recall their previous current moods. Our proposed alternative is that the aggregated retrospective evaluation of how one felt is a recalled weighted average of distorted memories of the impacts on mood of each evaluation. Thus, it is not the mood or the emotional response that is recalled, but each evaluation's impact on mood. In line with the judgment model proposed by Schwarz and Strack (1999), the aggregated evaluation may also be influenced by current mood at the time of recall. It follows from our proposition that distortions due to recall from memory would influence the aggregated mood impacts of evaluations of the events in the sequence. Reflecting the operation of the serial position effect in free recall of a sequence of to-be-remembered items (e.g., Davelaar et al., 2005), mood impacts on evaluations in the beginning (primacy) and end (recency) are expected to be more easily recalled.^3^ Furthermore, time decay may dampen the memory of the mood impacts on the evaluations. In the following model aggregated recalled impacts on mood (recalled mood impacts or *RMI*) at time *n*+1 are related to the mood impacts of evaluations of each of *n* events that evoked emotional responses,

(3) $$RMI_{n+1}=\{\begin{array}{l} \alpha CM_{n+1}+\left(1-\alpha \right)\left(\frac{1}{n}\right)\sum_{1}^{n}\gamma CM_{max}\\ \;\left(1-\exp\left(-\delta \phi_{i}IU_{i}\right)\right)\;IUi\geq 0\\ \alpha CM_{n+1}+\left(1-\alpha \right)\left(\frac{1}{n}\right)\sum_{1}^{n}\gamma CM_{min}\\ \;\left(1-\exp\left(\delta \phi_{i}IU_{I}\right)\right)\;IUi<0 \end{array}$$

$\begin{array}{l} \text{where }\gamma =\left(\beta {\left(\frac{i-1}{n-1}\right)}^{s}+\left(1-\beta \right){\left(\frac{n-i}{n-1}\right)}^{s}\right);0\alpha ,\beta \leq \\ 1;s\;>\;1;0\;<\;\delta \;<\\ \;\phi_{i}. \end{array}$

Note that the recall of the mood impacts on evaluations varies from 0 when *IU*_*i*_ = 0 or δ = 0 to *CM*_*max*_ = −*CM*_*min*_ > 0. The parameter α determines the degree of influence of current mood at the time of recall. The limiting cases are that only current mood has an influence, thus resulting in the same answer to the questions “How do I feel now?” as “How did I feel?” or that current mood has no influence. We propose that α increases with difficulty in retrieving from memory the mood impacts. If no impacts are retrieved, current mood is thus reported in response to the question of how one felt during the sequence. The parameter β in conjunction with the parameter *s* captures the weights placed on the beginning and end of the sequence. Here the limiting cases are that there will only be a beginning or only an end effect. The parameter δ is the dampening factor accounting for underestimation of the mood impacts of the evaluations.

## Theoretical predictions

In this paper, we report two experiments to investigate how current mood (*CM*) and recalled mood impacts (*RMI*) are differently influenced by a sequence of events that evoke emotional responses. We use short sequences of monetary outcomes (referred to as lottery outcomes) having a fixed probability of losing or winning different amounts of money. Since lottery outcomes are frequently used in studies of choice (e.g., Kahneman and Tversky, 2002), our events represent a natural operationalization of instant utility. Gehring and Willoughby (2002) also inferred from brain scans emotional responses to sequences of monetary gains and losses. Langer et al. (2005) used experienced monetary outcomes (referred to as a “consumption streams”) in a study of evaluations of sequences. In order to avoid that participants add the outcomes as they did in their experiments, participants were informed that they would receive one randomly chosen lottery outcome. We assume then that each lottery outcome evokes an emotional response that increases with its size, either positive or negative depending on whether it is a potential gain or loss.

**Figure 1:**
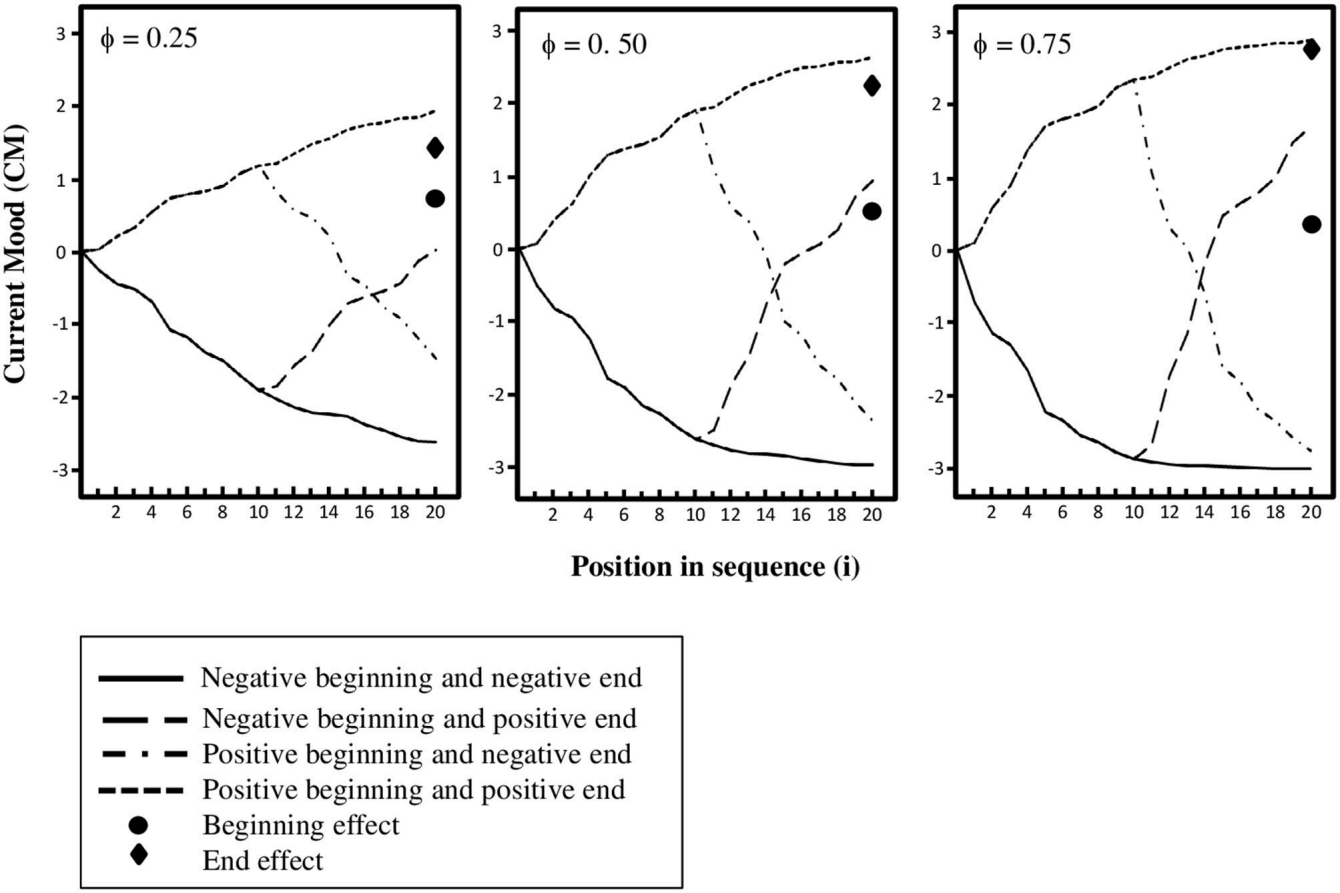
**Current mood (***CM***) plotted against position in the sequence of lottery outcomes (i) for three levels of mood impact (Φ)**. (Current mood is calculated by means of equation 1 with *CM*_0_ = 0, *CMMAX* = − *CMMIN* = 3, and η_*i*_ = 0, and equation 2 with *a*_*G*_ = *a*_*L*_/2 = 0.02 and *b* = 0.75. Note that the beginning and end effects at *i* = 20 are divided by 2 to not exceed the range of the Y-axis).

The sequences of lottery outcomes that we present in the experiments are shown in Table 1. In two sequences all 20 outcomes are losses (referred to as a negative beginning and a negative end), in two sequences 10 losses are followed by 10 gains (negative beginning and positive end), in two sequences 10 gains are followed by 10 losses (positive beginning and negative end), and in two sequences all 20 outcomes are gains (positive beginning and positive end). In order to show theoretically how current mood changes with the gains and losses in the different sequences, in Figure 1 current mood (*CM*_*i*_) calculated by means of equations 1 and 2 is plotted against order (*i*) in the sequence for three different levels of mood impact (ϕ_*i*_ > 0) that are constant for all *i*. We set *CM*_0_ = 0 and *CMIN* = −*CMAX* = 3 in equation 1, and *a*_*G*_ = *a*_*L*_/2 = 0.02 and *b* =.75 in equation 2.^4^ Averages are calculated across the two replicates of each of the different sequences in Table 1. As can be seen, the differences between the sequences with positive ends and those with negative ends (end effects) are larger than the differences between the sequences with positive beginnings and those with negative beginnings (beginning effects). We thus expect that there will be end effects that are larger than the beginning effects. It should also be noted that the differences between the end effects and beginning effects increase with the mood impact of the emotional responses.

**Table 1:** **Sequences of lottery outcomes (in SEK) presented in the experiments**.

	**Negative beginning**				**Positive beginning**			
	**Negative end**		**Positive end**		**Negative end**		**Positive end**	
**Position in sequence**	**1**	**2**	**1**	**2**	**1**	**2**	**1**	**2**
1	−27	−19	−19	−27	3	6	3	6
2	−30	−13	−13	−30	28	38	28	38
3	−19	−4	−4	−19	18	46	18	46
4	−2	−15	−15	−2	45	20	45	20
5	−8	−48	−48	−8	38	44	38	44
6	−41	−9	−9	−41	8	23	8	23
7	−32	−29	−29	−32	6	9	6	9
8	−49	−14	−14	−49	13	19	13	19
9	−39	−35	−35	−39	47	3	47	3
10	−4	−43	−43	−4	23	43	23	43
11	−19	−27	3	6	−19	−27	6	3
12	−13	−30	28	38	−13	−30	38	28
13	−4	−19	18	46	−4	−19	46	18
14	−15	−2	45	20	−15	−2	20	45
15	−48	−8	38	44	−48	−8	44	38
16	−9	−41	8	23	−9	−41	23	8
17	−29	−32	6	9	−29	−32	9	6
18	−14	−49	13	19	−14	−49	19	13
19	−35	−39	47	3	−35	−39	3	47
20	−43	−4	23	43	−43	−4	43	23

**Figure 2:**
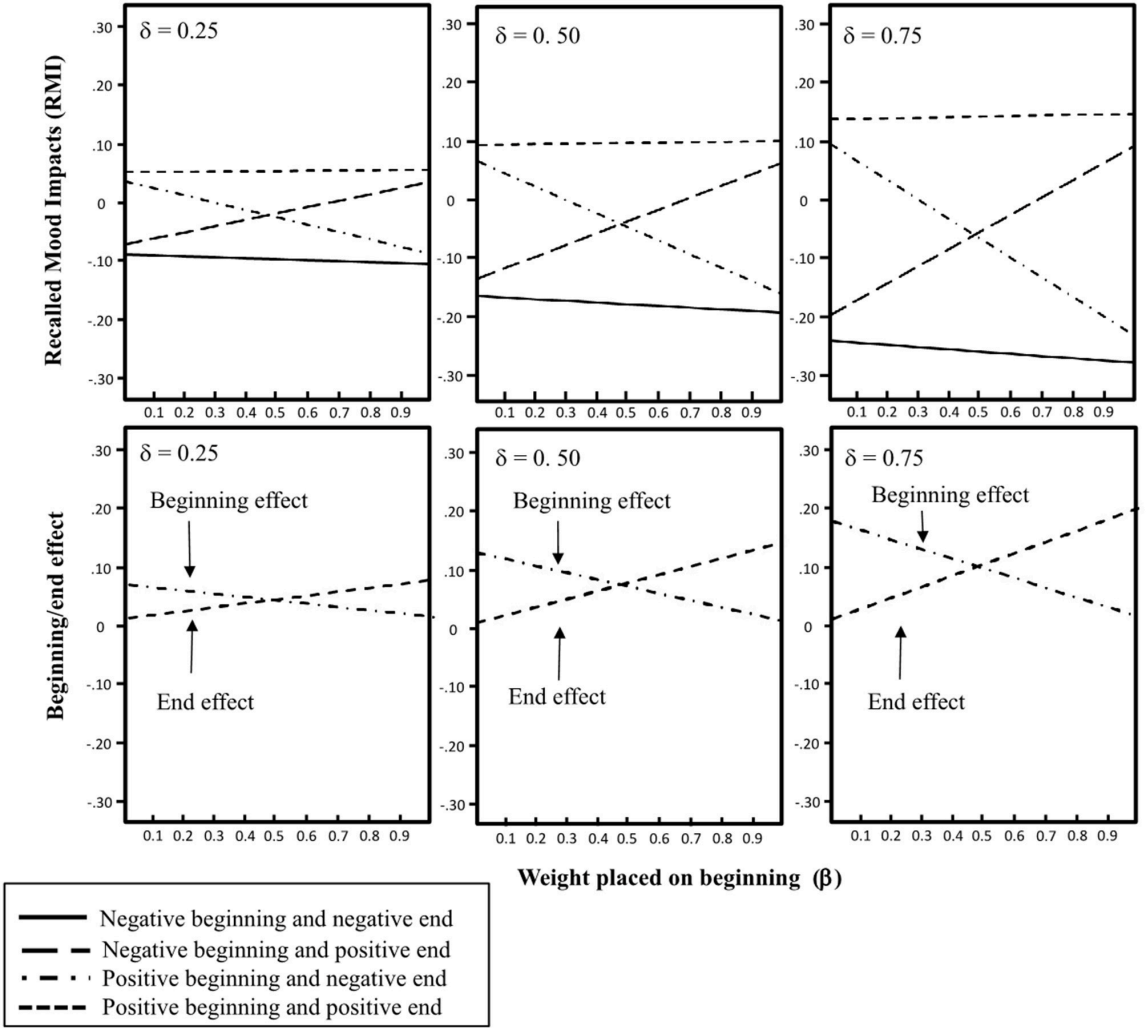
**Recalled mood impacts (***RMI***), beginning effect, and end effect plotted against weight placed on the beginning of the sequence of monetary outcomes (β) for three levels of memory decay (δ)**. (Recalled mood impacts is calculated by means of equation 3 with α = 0, *CM*_0_ = 0, and *CMMAX* = − *CMMIN* = 3, and equation 2 with *a*_*G*_ = *a*_*L*_/2 = 0.02, and *b* = 0.75).

We similarly make theoretical predictions of recalled mood impacts (*RMI*_*n*+1_) after the end of the sequence (*i* = *n* = 20). In Figure 2 *RMI*_*n*+1_ calculated by means of equation 3 (for α = 0, *s* = 2, *a*_*G*_ = *a*_*L*_/2 = 1, *b* = 0.75, and *CMIN* = −*CMAX* = 3) is plotted against β varying from 0 to 1 for three levels of dampening (δ). The beginning and end effects are below plotted against β. It can be seen that the size of the beginning and end effects vary.

Our theoretical results substantiated by the plots in Figures 1, 2 are that for the sequences of lottery outcomes in Table 1 current mood would always yield an end effect, whereas recalled mood impacts of the evaluations would yield both a beginning and an end effect given that both beginning and end are remembered. Based on these theoretical results we attempt to distinguish between current mood and recalled mood impacts by investigating conditions under which an end effect would be observed for current mood and a beginning and end effect for recalled mood effects.

## Experiment 1

In Experiment 1 we test the hypothesis that current mood is only influenced by the end of a sequence of lottery outcomes evoking emotional responses, whereas recalled mood impacts are influenced by both the beginning and end. In order to prevent thinking back on the lottery outcomes such that the ratings of how participants felt during the sequence would influence current mood, participants are asked to perform the allegedly unrelated task of rating how they feel when viewing a nature picture immediately after being presented the lottery outcomes. They are then asked to rate how they felt during the sequence. In a control condition other participants only rate how they felt during the sequence.

### Method

#### Participants

Participants were 87 undergraduates (50 women; mean age 23.55 years, standard deviation = 3.63 years, and range 18–36 years) at Karlstad University enrolled in different study programs. They volunteered to participate in compensation for SEK 50 (approximately equivalent to USD 8) for showing up and the opportunity of earning an additional SEK 100 as a lottery outcome. Between 20 and 24 participants were randomly assigned to each of four between-groups conditions.

Another 62 participants (45 women; mean age 24.18 years, standard deviation = 4.93 years, and range 19–40 years) from the same pool of undergraduates volunteered to participate for the same compensation in a control condition conducted separately. Between 15 and 16 participants were randomly assigned to the same four between-groups conditions.

#### Material

Each participant was presented with a sequence of 20 potential lottery outcomes (see Table 1), either 20 gains (positive beginning and end), 20 losses (negative beginning and end), 10 losses before 10 gains (negative beginning and positive end), or 10 gains before 10 losses (positive beginning and negative end). Two part-sequences consisting of 10 gains were obtained by random sampling without replacement of numbers in the interval 1 to 49 and another two part-sequences consisting of 10 losses were obtained by random sampling without replacement of numbers in the interval –1 to –49. In combining the part-sequences to full sequences (20 lottery outcomes), two replicates were constructed of each full sequence (two of each of 20 gains, 20 losses, 10 gains before 10 losses, and 10 losses before 10 gains). The replicates were presented about equally often across participants.

#### Experimental design

The experimental design was a randomized factorial with two between-groups factors (positive vs. negative valence of the beginning of the sequence X positive vs. negative valence of the end of the sequence) and one within-groups factor (ratings of current mood vs. recalled mood impacts). In the control condition the experimental design was replicated except that participants only rated recalled mood impacts.

#### Procedure

**Figure 3:**
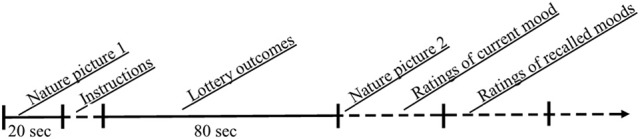
**The time sequence of activities in Experiment 1**. (Broken lines indicate that time was not fixed but dependent on when participants finished).

Participants serving in groups of 2 to 8 were seated in a room in front of a large screen. As shown in Figure 3 a nature picture^5^ was first shown on the screen for 20 s to minimize initial differences in current mood. The instructions were then presented as text on the screen as well as aurally from a loud speaker. Participants were told that they were endowed with SEK 50 as a buy-in to a lottery before being presented the following instructions (translated from the Swedish): “*You will now be presented a sequence of potential lottery outcomes. As will be shown on the screen, the sequence consists of a number of chances to increase or decrease the SEK 50 you have been given*. One *lottery outcome will be randomly selected after that all have been presented. Your endowment of SEK 50 may increase to SEK 100 or decrease to SEK 0 depending on which lottery outcome that is selected. After the sequence you will be asked to answer a number of questions*.”

**Figure 4:**
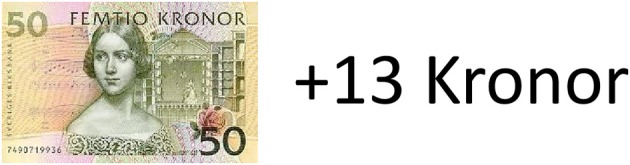
**An example of the lottery outcomes presented to the participants**.

After the instructions had been presented, the lottery outcomes were each presented for 4 s appearing on the screen as if being added to or deducted from the endowment of SEK 50 (represented by a picture of a 50-SEK note^6^) (see Figure 4), for instance SEK 50 + SEK 10, or as being deducted from SEK 50, for instance SEK 50–SEK 10.

After that the lottery outcomes had been presented, participants turned to the first page in a booklet on a table in front of them and read the instructions printed on this page. Another nature picture was shown for 8 s while participants rated how they felt (current mood). The ratings were made on four 7-point unipolar scales appearing on the next page of the booklet. The scales that ranged from 0 (not at all) to 6 (extremely much) were defined by the adjectives glad, sad, active, and passive. Thereafter, when the picture was no longer shown, participants turned to the next page in the booklet and rated on another set of identical scales how they had felt during the lottery outcomes (recalled mood impacts). The instructions read “*Think back about the lottery outcomes and rate on the scales how you felt when you saw them.”* In the control condition no nature picture was shown after the sequence of lottery outcomes and participants were only asked to rate how they felt during the lottery outcomes.

At the end of the session questions were asked about participants' experiences of the lottery outcomes followed by background questions. Participants were finally informed about which lottery outcome was selected. In connection with debriefing, all participants were then paid their compensation of SEK 50 for showing up and an additional SEK 100 if the lottery outcome was a gain. Sessions lasted for approximately 15 min.

### Results

The ratings were averaged across all four adjective scales after reversing the 0-to-6 scale ratings of sad and passive to 6-to-0 scales (*Y* = 6 − *X*).^7^ This resulted in Cronbach's αs of 0.645 for the average ratings of current mood and 0.707 and 0.742 (control condition) for the average recalled mood impacts. A transformation was then made to -3-to-3 scales. Means and standard deviations are given in Table 2 related to the valence (gain or loss) of the beginning and end of the sequences of lottery outcomes.

**Table 2:** **Mean ratings (and standard deviations) of current mood and recalled mood impacts related to negative vs. positive beginning and negative vs. positive end (experiment 1)**.

	**Negative beginning**				**Positive beginning**			
	**Negative end**		**Positive end**		**Negative end**		**Positive end**	
	**M**	**(Sd)**	**M**	**(Sd)**	**M**	**(Sd)**	**M**	**(Sd)**
Ratings of current mood	0.25	(1.37)	0.97	(0.81)	0.38	(1.00)	0.99	(0.98)
Ratings of recalled mood impacts after current mood	−0.10	(1.90)	0.50	(1.34)	0.07	(1.53)	0.80	(1.58)
Ratings of recalled mood impacts independent of current mood (control condition)	−0.33	(1.21)	0.11	(1.46)	0.23	(1.38)	0.28	(1.33)

*The ratings vary from −3 to 3*.

An end effect (0.66) and no beginning effect (−0.08) are as expected observed for the ratings of current mood,^8^ whereas both a beginning and an end effect (0.24 and 0.67; and in the control condition 0.37 and 0.24) are observed for the ratings of recalled mood impacts. However, a 2 (beginning: Positive vs. negative) X 2 (end: Positive vs. negative) X 2 (ratings: Current mood vs. recalled mood impacts) analysis of variance (ANOVA) with repeated measures on the last factor excluding the control condition only yielded significant main effects of the ratings (*M*_*current mood*_ = 0.65 vs. *M*_*recalled mood impacts*_ = 0.32), *F*_(1, 83)_ = 5.21, *p* = 0.025, partial ω^2^ = 0.046, and of the end (*M*_*positive end*_ = 0.81 vs. *M*_*negative end*_ = 0.15), *F*_(1, 83)_ = 7.10, *p* = 0.009, partial ω^2^ = 0.066.

**Figure 5:**
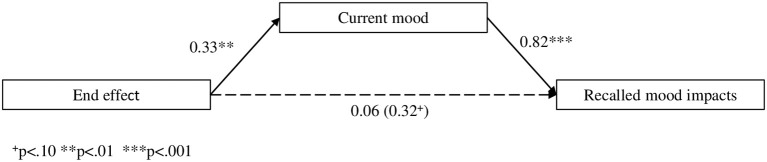
**Unstandardized regression coefficients from mediation analysis (Experiment 1)**.

The expected three-way interaction between ratings and beginning and end did thus not reach significance, *F*_(1, 83)_ < 1. A possible reason is the influence of current mood on the ratings of mood impacts. In order to substantiate this interpretation, a mediation analysis (MacKinnon, 2008; Zhao et al., 2014) was performed. The correlation between current mood and recalled mood impacts was overall .566 (*p* < 0.001), varying between 0.443 and 0.606 in the different conditions. In linear regression analyses end had a significant effect on current mood (*b* = 0.33, *SE* = 0.11, *p* = 0.004) and a marginally significant effect on recalled mood impacts (*b* = 0.32, *SE* = 0.17, *p* = 0.057). Current mood was significant (*b* = 0.82, *SE* = 0.14, *p* < 0.001) when entered in a regression analysis on recalled mood impacts, whereas the end effect did not remain significant (*b* = 0.06, *SE* = 0.15, *p* = 0.713). In a Sobel test the indirect effect was significant, *z* = 2.65, *p* < 0.008, and as Figure 5 shows, current mood fully mediates the end effect on recalled mood impacts.

An additional 2 (ratings: Recall mood impacts after ratings of current mood vs. no ratings of current mood) X 2 (beginning: Positive vs. negative) X 2 (end: Positive vs. negative) ANOVA was performed on recalled mood impacts including the control condition. All the interactions with ratings were non-significant (*F*s < 1), thus failing to find significant effects of whether the ratings of recalled mood impacts were made after the mood ratings or if no ratings of current mood were made. The main effect of the end approached significance, *F*_(1, 143)_ = 3.44, *p* = 0.066, partial ω^2^ = 0.016, whereas the main effect of the beginning marginally failed to reach significance, *F*_(1, 143)_ = 2.12, *p* = 0.120, partial ω^2^ = 0.007.

### Discussion

The results showed as expected from equations 1 and 2 an end effect on the ratings of current mood. Not as expected however, no interaction with ratings was significant suggesting that there was no beginning effect on the ratings of recalled mood impacts. One reason for this may be that, as specified in equation 3 and substantiated by the mediation analysis, current mood influenced recalled mood impacts. Yet, the beginning effect was larger for recalled mood impacts than current mood. Statistically, the results in the control condition did not differ. It is still noteworthy that in the control condition the end effect was weaker as would be expected if there is no influence of current mood.

The significant overall difference between the ratings of current mood and recalled mood impacts appears to be inconsistent with that the former is dependent on the latter. One possible explanation is that the positive current mood was inflated similarly for all participants when they viewed the nature picture and then reverted back when the picture was no longer shown during recall of the mood impacts. For this reason then, recalled mood impacts after the ratings of current mood were presumably influenced by this reduced current mood. Furthermore, in the absence of the ratings of current mood, recalled mood impacts were presumably for the same reason lower when no nature picture was shown.

It is possible that the mediation of current mood on recalled mood impacts is due to a failure to recall the mood impacts. Thus, the ratings of current mood were substituted for recall of mood impacts when not possible or difficult to recall.

## Experiment 2

In Experiment 2 we investigate whether improved memory of the sequence of lottery outcomes would result in a beginning and end effect in recalled emotion impacts. We first present a sequence of monetary amounts without disclosing that they are lottery outcomes until after their presentation. If the monetary amounts are not perceived as gains or losses, no effects on the ratings of current mood are expected before the disclosure. After the disclosure and if the participants remember the beginning and end of the sequence, we expect beginning and effects on the ratings of recalled mood impacts.

### Method

#### Participants

Another 41 undergraduates (19 women; mean age 25.9 years, standard deviation = 6.2, and range 20–48 years) at Karlstad University volunteered to participate in compensation for SEK 50 for showing up and being promised a performance-dependent additional maximal sum of SEK 100. Twenty participants were randomly assigned to a “positive beginning—negative end” condition and 21 participants randomly assigned to a “negative beginning—positive end” condition.

#### Procedure

The sequences of lottery outcomes (instead referred to as monetary amounts) consisted of the two replicates presented in Table 1 with a negative beginning followed by a positive end and the two replicates with a positive beginning followed by a negative end. The procedure was the same as in the main condition of Experiment 1, with two mood ratings and the presentation of a nature picture before and after the sequence of monetary amounts. In the instructions participants were told that they would later be asked to remember the monetary amounts. They were further told that they would have SEK 50 as a buy-in to a lottery after the recall task. Following the sequence of monetary outcomes, participants were asked to rate how they feel (current mood) while watching the second nature picture. The instructions and rating scales were the same as in the preceding experiment. When the ratings of current mood had been made and the nature picture was no longer shown, additional written instructions informed participants that one monetary amount of those presented would be randomly selected such that their endowment of SEK 50 may increase to maximally SEK 100 or be reduced to minimally SEK 0 depending on which monetary amount was selected. They were then asked to rate on the same scales how they felt (recalled mood impacts) when thinking about the monetary amounts. Participants were finally asked to recall the monetary amounts in any order. Their answers were recorded on an answer sheet with 30 unnumbered lines.

The sessions lasted for about 15 min including the same post-experimental questions as in the preceding experiment, announcing and paying the compensation (SEK 100 to everyone), and debriefing.

### Results and discussion

Table 3 displays for each sequence of monetary amounts means and standard deviations of the ratings of current mood and of recalled mood impacts averaged as in the preceding experiment. Cronbach αs were 0.639 for the average ratings of current mood and 0.748 for the average ratings of recalled mood impacts. Mean number recalled gains and losses are also shown in the table. Each recalled monetary amount below SEK 50 was scored as a loss and each amount above SEK 50 as a gain.

**Table 3:** **Mean ratings (and standard deviations) of current mood and recalled mood impacts and mean number recalled gain and loss lottery outcomes related to sequences with negative beginning and positive end and positive beginning and negative end (experiment 2)**.

	**Negative beginning and positive end**		**Positive beginning and negative end**	
	**M**	**(Sd)**	**M**	**(Sd)**
Ratings of current mood	−0.08	(1.08)	0.16	(1.18)
Ratings of mood impacts	−0.40	(1.18)	0.41	(1.12)
Number of recalled gains	3.81	(1.21)	5.55	(1.93)
Number of recalled losses	4.62	(2.84)	2.65	(1.69)

*The ratings vary from −3 to 3*.

In support of the hypothesis, the results differed for the ratings of current mood and recalled mood impacts. A 2 (ratings: Current mood vs. recalled mood impacts) X 2 (sequence: Positive beginning and negative end vs. negative beginning and positive end) ANOVA yielded no significant main effects, *F*_(1, 39)_ < 1, for ratings, and *F*_(1, 39)_ = 2.65, *p* = 0.112, partial ω^2^ = 0.039, for sequence, and a marginally significant interaction between ratings and sequence, *F*_(1, 39)_ = 4.04, *p* = 0.051, partial ω^2^ = 0.069. As expected, current mood did not change but recalled emotion impacts did. In an independent-samples *t*-test the sequence of monetary amounts had no significant effect on the ratings of current mood, *t*_(39)_ < 1. This should be compared to the results of Experiment 1 reported in Table 2 yielding a large difference between the same two sequences (*M*_*negative beginning*/*positive end*_ = 0.97 vs. *M*_*positive beginning*/*negative end*_ = 0.38). In contrast, sequence had an expected significant effect on the ratings of recalled mood impacts, *t*_(39)_ = 2.28, *p* = 0.028, *d* = 0.730. Its direction suggests it is a beginning effect.

That sequence has a beginning effect on recalled mood impacts is also supported by that more gains are recalled when presented first in the sequences and recalled mood impacts is positive, but that more losses are recalled when presented first in the sequences and recalled mood impacts are negative. A (sequence of monetary amounts: Positive beginning and negative end vs. negative beginning and positive end) X 2 (recall: Gains vs. losses) ANOVA showed that both the main effect of gains vs. losses and the interaction between gains vs. losses and sequence were significant, *F*_(1, 39)_ = 8.86, *p* = 0.005, partial ω^2^ = 0.161, and *F*_(1, 39)_ = 27.90, *p* < 0.001, partial ω^2^ = 0.396. When gains precede losses in the sequence of monetary amounts, independent-samples *t*-tests showed that significantly more gains than losses are recalled, *t*_(39)_ = 2.54, *p* = 0.015, *d* = 0.813, and when losses precede gains, that significantly more losses than gains are recalled, *t*_(39)_ = 2.68, *p* = 0.011, *d* = 0.858.

The results support the hypothesis that monetary amounts fail to change current mood if not identified as gains or losses such that they do not evoke emotional responses. In contrast, when monetary amounts are retrospectively identified as lottery outcomes, the mood impacts of the gains and losses appearing in the beginning of the sequence are recalled, which results in a beginning effect on recalled mood impacts. The interference and delay caused by the ratings of current mood likely suppressed an end effect on recall and thus an end effect on recalled mood impacts.

## General discussion

In comparing the results of Experiments 1 and 2 we argue that support is obtained for the distinction between evaluations and emotional responses in that the monetary amounts in the sequence were found to have impacts changing current mood only when perceived as gains or losses. We further argue that the results of the experiments are consistent with (i) that current mood as specified in equations 1 and 2 is updated during the sequence of gains or losses that evokes emotional responses, and (ii) that recalled mood impacts as specified in equation 3 is influenced by current mood at recall as well as by the beginning of the sequence of mood impacts if it is remembered.

We theoretically expected to observe both beginning and end effects on recalled mood impacts. In Experiment 1 we found the expected end (recency) effect on current mood and recalled mood impacts, but no statistically significant expected beginning (primacy) effect on recalled mood impacts. The estimated beginning effect was however larger for recalled mood impacts than for current mood, in particular in the control condition when participants did not rate their current mood. In Experiment 2 only a beginning effect and no end effect was observed on recalled mood impacts. If assuming that the beginning effect depends on storage and retrieval from long-term memory and the end effect on recall from short-term memory (Davelaar et al., 2005), a possible account of the result is that in both experiments the preceding ratings of current mood interfered with recall from short-term memory. In Experiment 1 this resulted in a dependency on current mood, presumably because also long-term memory retrieval of the beginning was impaired without explicit instructions to memorize the gains and losses, which however was not the case in Experiment 2 when participants were asked to remember the monetary amounts. The account is further supported by the weaker end effect in the control condition of Experiment 1 when no ratings of current mood were made. We acknowledge that this interpretation, although consistent with the results, is not the only possible and that the support for it is not strong. Conceptual replications would be highly valuable.

Because we did not measure current mood during the sequence of lottery outcomes presented to the participants in the experiments,^9^ inferences are made from the observed end effect in Experiment 1. This is consistent with the theoretical predictions that an end effect should be expected if the outcomes in the sequence have impacts on current mood. An end effect is a common finding in previous research examining evaluations of sequences of emotion stimuli in that linear increases are more positive than linear decreases (Hsee et al., 1991; Ariely, 1998; Chapman, 2000), and that accelerating increases are more positive than linear increases whereas accelerating decreases are less positive than linear decreases (Hsee and Abelson, 1991; Hsee et al., 1994; Ariely, 1998).

That current mood is updated in response to evaluations of events that evoke emotional responses is a conceptualization of how sequences of events have impacts that differs from the notion of remembered utility proposed in previous research (Kahneman, 2000a,b). An important difference is that we make explicit the distinction between evaluation and emotional response. In the same vein Zauberman et al. (2006) argued that a distinction should be made between hedonic and informational descriptive or predictive evaluations. In three experiments with different stimuli (test performance, defective products from a factory, service quality) with increasing or decreasing sequences, they found consistent end effects (increasing sequences evaluated as more positive than decreasing sequences) for hedonic evaluations but only for predictive informational evaluations. Yet, our experiments differed from those of Zauberman et al. (2006) in that we asked participants to rate how they themselves felt at the moment when they were viewing a nature picture instead of how satisfied they believe another person would be with the sequence. In our experiments the increasing and decreasing sequences did not follow a trend but a step function. Our sequences also made possible to obtain independent estimates of beginning and end effects.

The explanation proposed by Zauberman et al. (2006) (see also Ariely and Carmon, 2000) is that expectation based on trend extrapolation plays an important role. In support of the role of expectation, in a recent study of mood changes during repeated choices of gambles, Rutledge et al. (2014) found that expectations played a more important role than a decaying memory of the accumulated outcomes. Explanations based on expectation and updating of current mood may however not be incompatible. Whereas, our explanation focuses on how one feels after a sequence of events, both how one feel and expectations about how one will feel in the future may coexist. A question this raises is what role the past plays?

Awareness of what causes changes in current mood is presumably low and updating is not likely to impose a substantial memory load (Van Dillen and Koole, 2007; Mikels et al., 2008). For this reason current mood may reflect the impact of previous events without these being remembered. Trend extrapolation may be a similar process of recurrent updating that results in an expectation of the future without any memory of the past. Making these assumptions does of course not rule out the possibility that some information is remembered. The peak-end rule (Fredrickson, 2000) is clearly a candidate conceptualization of what is remembered. Memory decay resulting in the serial position effect (Davelaar et al., 2005) is another.

An objection to our proposition that emotional responses change current mood is that it would seem to imply that current mood is highly variable. This is not consistent with the findings of previous research (e.g., Diener and Lucas, 2000; Diener et al., 2015). However, we propose that the impacts of emotional responses are dampened, thus current mood varies less. Furthermore, we do not propose that all events evoke emotional responses. In real life such events may not occur frequently. Current moods are therefore likely to be in general relatively stable.

A number of questions remain to be addressed, theoretically as well as empirically. A first issue concerns the validity of the distinction between evaluation and emotional response. It may seem more plausible to apply the distinction to instant utilities (Kahneman, 1999) which relies on no or minimal memory retrieval. Although extended in time, we still claim that updating is an automatic process minimally dependent on memory similar to the formation of an instant utility. Additional research is needed to further investigate this.

The trajectory of updated current mood may also be affected by changes in internal states (Russell, 2003, 2014; Damasio, 2010), for instance time-dependent changes due to fatigue. Another change is adaptation resulting in mood reverting to a positive set-point (Diener et al., 2015). An extended model of the updating of current mood would need to take this into account. Another short-coming would seem to be that we do not model expectations (Rutledge et al., 2014).

It is generally recognized (Isen, 2000) that emotion responses not only influence current mood but are also influenced by current mood. Thus, there is possibly a reverse direction of influence than proposed here. This would still be captured by the parameters in equation 1, although their interpretation needs to be extended.

A final issue is what determines a decision to repeat or not repeat a choice resulting in the sequence of outcomes. It has been argued that remembered utility is such a determinant (Kahneman, 2000a,b; Hsee and Hastie, 2006). We speculate that current mood after a sequence of choice outcomes may be another determinant of an immediate repeat decision but not to the same extent if the repeat decision is delayed. Current mood is also only one input to a deliberation process that eventually results in a decision (Damasio, 2010).

## Ethics statement

The research were conducted in accordance with approved research protocols. The procedure ensured that participants were informed that confidentiality were maintained. Before giving their consent to participate in the study, all participants were (1) informed about the procedure and participation, (2) that it was volunteer, (3) that they had the right to end their participation at any time without giving any reson for it. No vulnerable populations were involved in this study.

## Author contributions

All authors have made substantial, direct and intellectual contribution to the work, and approved it for publication.

## Funding

The work was supported by VINNOVA - the Swedish Governmental Agency for Innovation Systems - under Grant 2014-05335.

### Conflict of interest statement

The authors declare that the research was conducted in the absence of any commercial or financial relationships that could be construed as a potential conflict of interest.

## Footnotes

^1^According to several emotion theories (e.g., Carver and Scheier, 1990; Lazarus, 1991; Oatley, 2009), an emotional response (also referred to as emotion, emotion episode, or feeling) is evoked if the evaluation of an event has personal relevance, for instance, that attainment of a positive personal consequence is facilitated or a negative personal consequence avoided (Carver, 2001). Whether or not this is a necessary condition is not essential here. Yet, the distinction between evaluation and emotional response is important. It would be unrealistic and inconsistent with previous research (e.g., Diener et al., 2015) that every good-bad evaluation would evoke emotional responses.

^2^We posit theoretically an upper limit to a positive mood and a lower limit to a negative mood. In a similar vein Linville and Fischer (1991) suggested that people have limited resources to savour positive feelings and to suffer from negative feelings.

^3^In general, it is assumed that in free recall primacy reflects retrieval of information stored in long-term memory and recency information being held in short-term memory at the time of recall (Davelaar et al., 2005).

^4^The values of the parameters *a*_*B*,_*a*_*G*,_ and *b* are selected to be approximately consistent with the findings in a vast number of empirical studies (e.g. Tversky and Kahneman, 1992; McBride, 2010). Setting *CMIN* = −*CMAX* = 3 is consistent with the boundaries of the rating scale used in the experiments.

^5^Nature views have in several studies (e.g., Hartig et al., 2003) been shown to influence feelings.

^6^A picture of the note was used because previous research (Raghubir, 2006) suggests that a note may evoke a stronger emotional response than only the monetary amount presented in digits.

^7^We aggregated all the ratings to obtain a single measure varying from positive valence and high activation (elation) to negative valence and low activation (disappointment) (Västfjäll and Gärling, 2002, 2007). Aggregation was justified by the acceptable sizes of the Cronbach's *a*s.

^8^The beginning effect is calculated as the difference between the means of the sequences with positive beginnings and those with negative beginnings, whereas the end effect is calculated as the difference between the means of the sequences with positive ends and those with negative ends.

^9^Repeatedly measuring current mood by means of ratings during the sequence of potential lottery outcomes would not have been easily tractable or would possibly have had disruptive effects (Ariely, 1998; Ariely and Zauberman, 2000, 2003).
